# T2-Weighted Cardiac Magnetic Resonance Imaging of Edema in Myocardial Diseases

**DOI:** 10.1100/2012/194069

**Published:** 2012-09-02

**Authors:** Yasuo Amano, Masaki Tachi, Hitomi Tani, Kyoichi Mizuno, Yasuhiro Kobayashi, Shinichiro Kumita

**Affiliations:** ^1^Department of Radiology, Nippon Medical School, 1-1-5 Senadagi, Bunkyo-ku, Tokyo 113-8603, Japan; ^2^Department of the 1st Internal Medicine, Nippon Medical School, 1-1-5 Senadagi, Bunkyo-ku, Tokyo 113-8603, Japan

## Abstract

The purpose of this paper is to describe imaging techniques and findings of T2-weighted magnetic resonance imaging (MRI) of edema in myocardial diseases. T2-weighted cardiac MRI is acquired by combining acceleration techniques with motion and signal suppression techniques. The MRI findings should be interpreted based on coronary artery supply, intramural distribution, and comparison with delayed-enhancement MRI. In acute myocardial diseases, such as acute myocardial infarction and myocarditis, the edema is larger than myocardial scarring, whereas the edema can be smaller than the scarring in some types of nonischemic cardiomyopathy, including hypertrophic cardiomyopathy. T2-weighted MRI of edema identifies myocardial edema associated with ischemia, inflammation, vasculitis, or intervention in the myocardium and provides information complementary to delayed-enhancement MRI.

## 1. Introduction

Delayed-enhancement magnetic resonance imaging (MRI) is valuable for the diagnosis and assessment of the severity of both ischemic and nonischemic myocardial diseases [1–8]. However, delayed-enhancement MRI cannot necessarily distinguish between the myocardial diseases at the acute and chronic stages. T2-weighted cardiac MRI has been successfully applied to detect myocardial edema, which may be related to chest pain, fever, electrocardiogram (ECG) abnormalities, and increases in myocardial enzymes in the blood, in acute myocardial diseases [9–12]. The T2-weighted MRI, therefore, can give information about the myocardial diseases, which is complementary to delayed-enhancement MRI. The aim of this paper is to describe the imaging techniques of T2-weighted cardiac MRI of edema and its interpretation in myocardial diseases.

## 2. Imaging Techniques Used in T2-Weighted**** Cardiac MRI of Edema

T2-weighted cardiac MRI of edema is acquired by combining acceleration techniques with motion suppression and prepulse techniques. These MRI techniques freeze the cardiac and respiratory motion effectively with giving high contrast between the blood, fat, normal myocardium, and myocardial edema. 

### 2.1. Acceleration Techniques

Turbo spin-echo imaging with multiple refocusing pulses has replaced spin-echo imaging in T2-weighted cardiac MRI because the scan time is reduced by a factor of 10–12 [13]. A parallel imaging technique is also used to reduce the scan time [14, 15].

### 2.2. Motion Suppression Techniques

An ECG-gating technique is usually used for cardiac MRI. This technique allows for data acquisition at the end diastole when the myocardium is static. A breath-hold technique suppresses respiratory artifacts. Alternative methods to the breath-holding technique are navigator-gating and respiratory-gating techniques [16]. 

### 2.3. Prepulse Techniques

The black-blood prepulse technique, consisting of two inversion-recovery pulses combined with ECG-gating, is applied to T2-weighted cardiac MRI [17]. By using slice nonselective and selective 180° pulses, the static tissues experience net zero rotation, whereas the blood signal is nullified at the imaging slice. The black-blood prepulse technique suppresses the blood signal in the cardiac chamber, thereby improving the visualization of cardiac structures and myocardial edema. Fat-suppression technique using inversion-recovery or spectrally selective pulse highlights myocardial edema by reducing the signal of the adipose tissue close to the myocardium [13].

### 2.4. Quantitative Techniques

**Figure 1:**
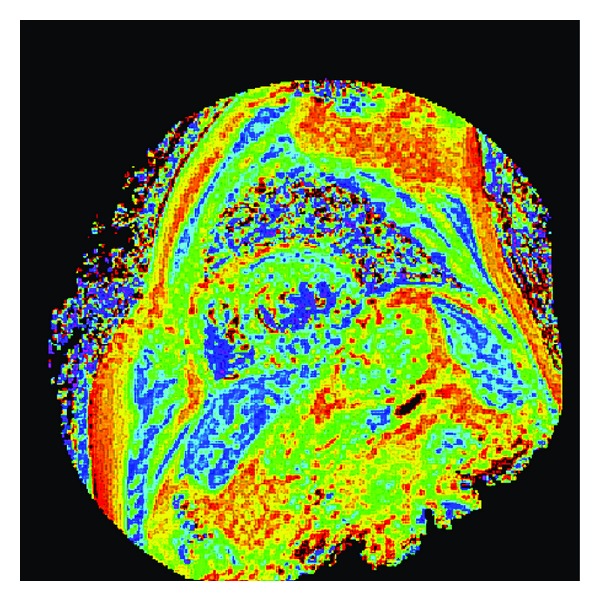
Myocardial T2 mapping generated from multiecho T2-weighted imaging.

Myocardial edema is quantified with T2-weighted MRI with T2-prepared or multiecho acquisition [18]. Zagrosek et al. [19] have reported that the measurement of the signal ratio between the myocardium and skeletal muscle is useful for detection of myocardial edema related to the irreversible myocardial injuries in acute myocarditis. However, in the current clinical routine, the multicoil and parallel imaging techniques are used, prohibiting the accurate measurement of the signal intensity of the tissues. Therefore, the T2-value measurement is more accurate and preferable when evaluating the myocardial edema quantitatively. T2 mapping generated from the T2-value measurement of the ventricular myocardium can allow for both visual and quantitative analysis of the myocardial edema (Figure 1).

## 3. Image Interpretation of T2-Weighted Cardiac MRI of Edema 

Image interpretation of T2-weighted cardiac MRI is based on comparison with coronary artery supply, intramural distribution (i.e., subendocardial, mesocardial, subepicardial) and morphology (e.g., mural, patchy, linear) of the edema, and comparison with delayed-enhancement MRI.

### 3.1. Distribution and Morphology of Myocardial Edema on T2-Weighted MRI 

**Figure 2:**
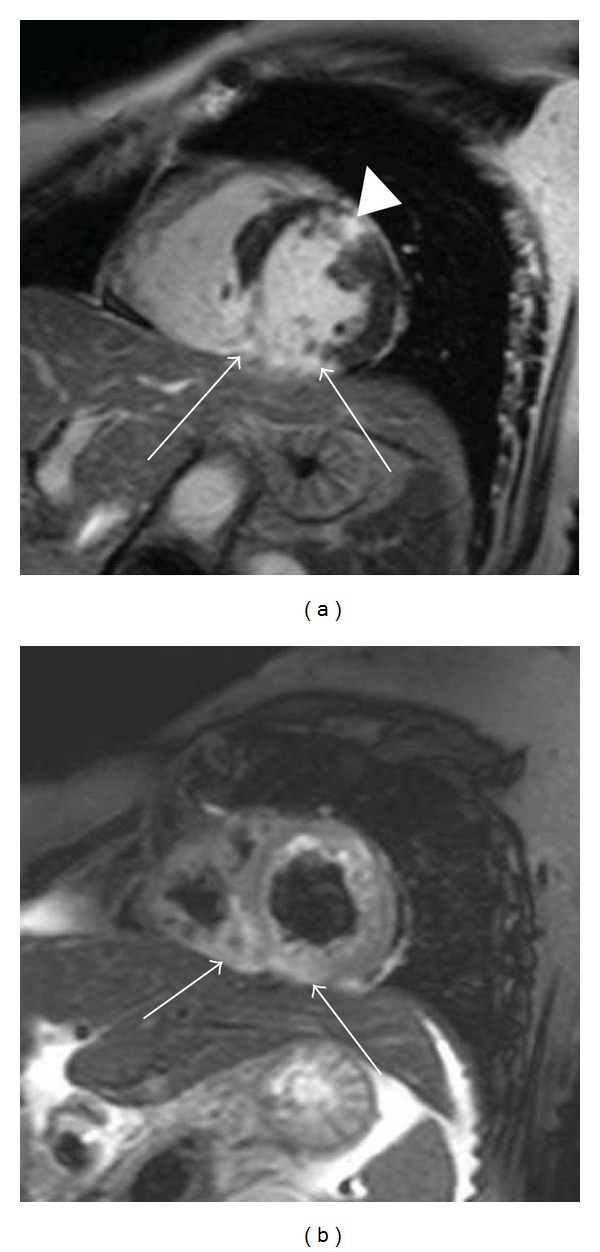
63-year-old female with myocardial infarction. Short-axis delayed-enhancement MRI (a) shows myocardial infarction at the inferior myocardium (arrows) and the anterior myocardium (arrowhead). Short-axis T2-weighted cardiac MRI (b) shows only myocardial edema associated with acute myocardial infarction in the inferior myocardium and right ventricular myocardium (arrows) that are consistent with blood supply from the right coronary artery.

**Figure 3:**
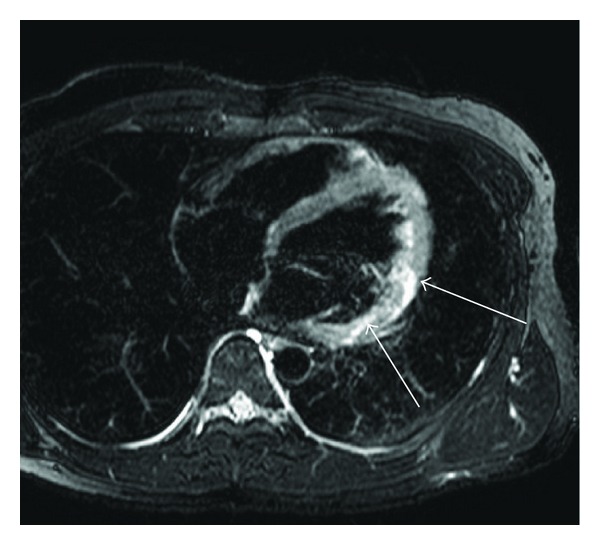
25-year-old male with acute myocarditis. T2-weighted MRI shows myocardial edema associated with acute myocarditis (arrows). The edema predominantly involves the epicardial or transmural myocardium in the lateral wall.

**Figure 4:**
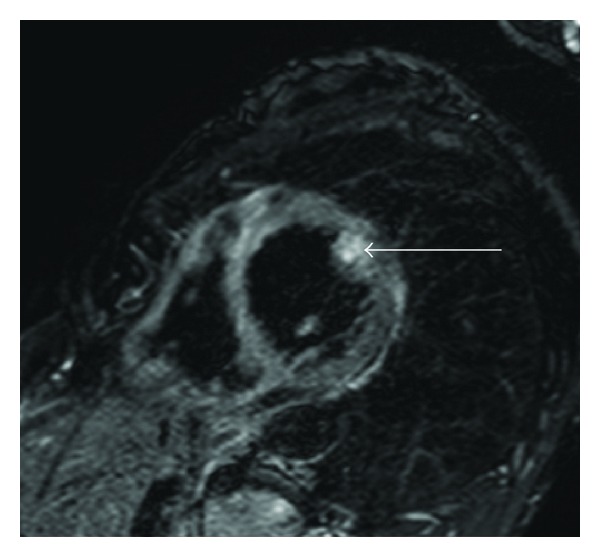
50-year-old female with Churg-Strauss syndrome. Short-axis T2-weighted MRI shows patchy myocardial edema of the papillary muscle (arrow) in the eosinophilic myocarditis associated with this syndrome.

**Figure 5:**
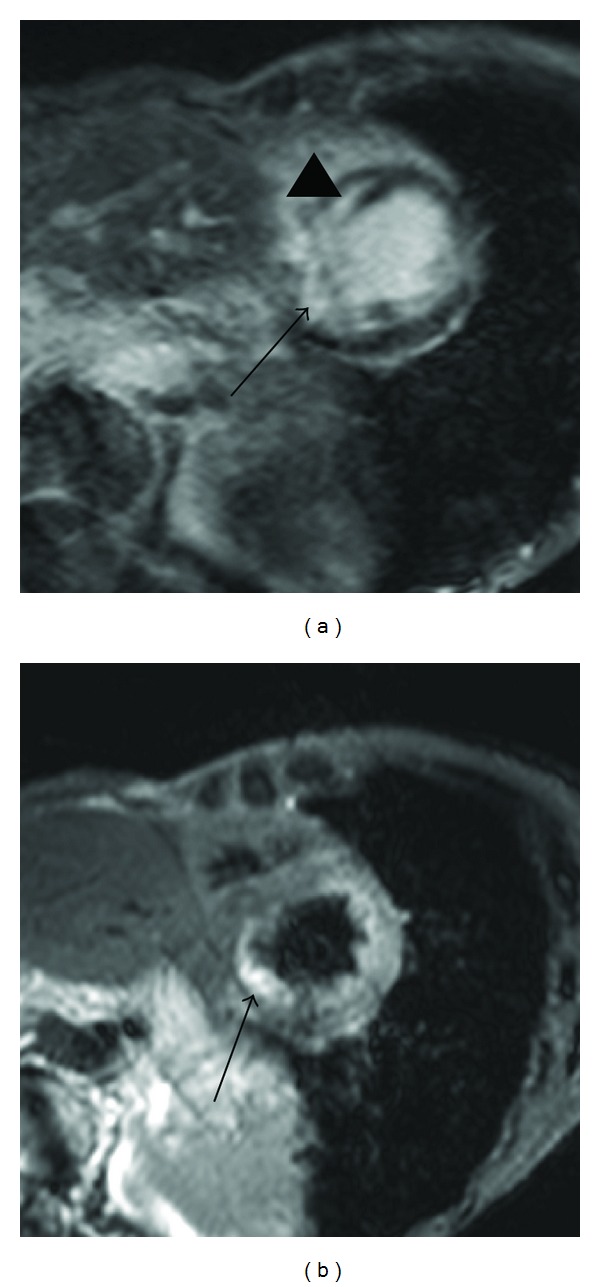
73-year-old female with cardiac sarcoidosis presenting with lower ejection fraction and conduction disturbance. Short-axis delayed-enhancement MRI (a) shows abnormal enhancement consistent with the edema (arrow) as well as the septal mesocardial myocardial scarring (arrowhead). T2-weighted cardiac MRI (b) shows myocardial edema at the transmural myocardium of the inferior region (arrow).

**Figure 6:**
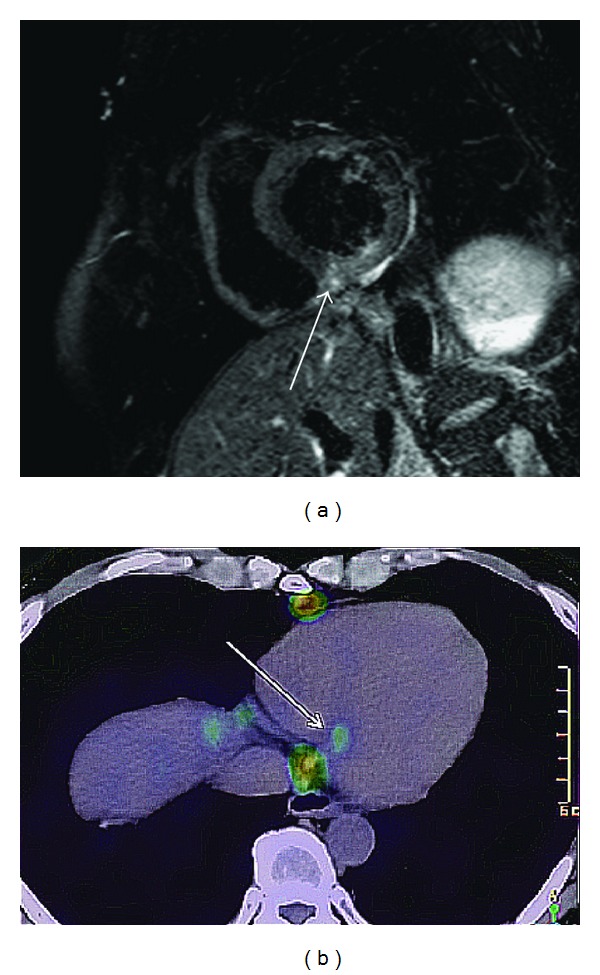
41-year-old male with cardiac sarcoidosis presenting with premature ventricular contraction. Short-axis T2-weighted MRI (a) shows a small myocardial edema at the inferior septal myocardium (arrow). ^18^FDG-PET (b) shows the focal inflammation (arrow), consistent with the edema on MRI.

**Figure 7:**
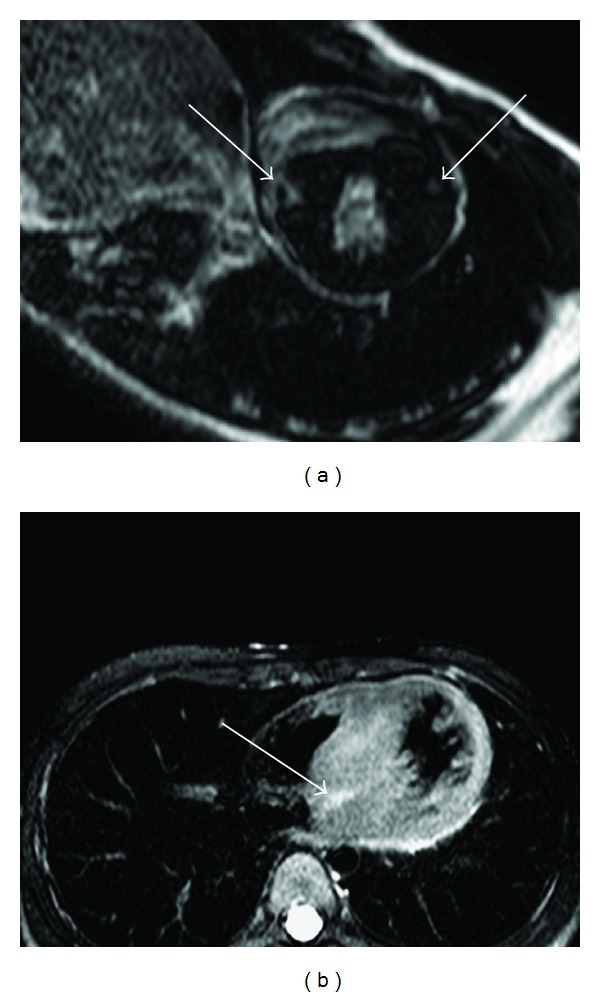
77-year-old female with hypertrophic cardiomyopathy presenting with chest pain. Delayed-enhancement MRI (a) shows patchy enhancement at the anterior and inferior septal myocardium (arrows). T2-weighted cardiac MRI (b) shows myocardial edema at the inferior and inferior septal myocardium (arrow).

The location of the myocardial edema should be compared with the coronary artery supply. Myocardial edema or ischemia associated with acute myocardial infarction distribute to the coronary artery supply and often show transmural involvement (Figure 2). The myocardial edema associated with nonischemic cardiomyopathy tends to localize in the mesocardial and subepicardial myocardium and appears patchy (Figures 3–5, 6(a), and 7).

### 3.2. Comparison with Delayed-Enhancement MRI

**Figure 8:**
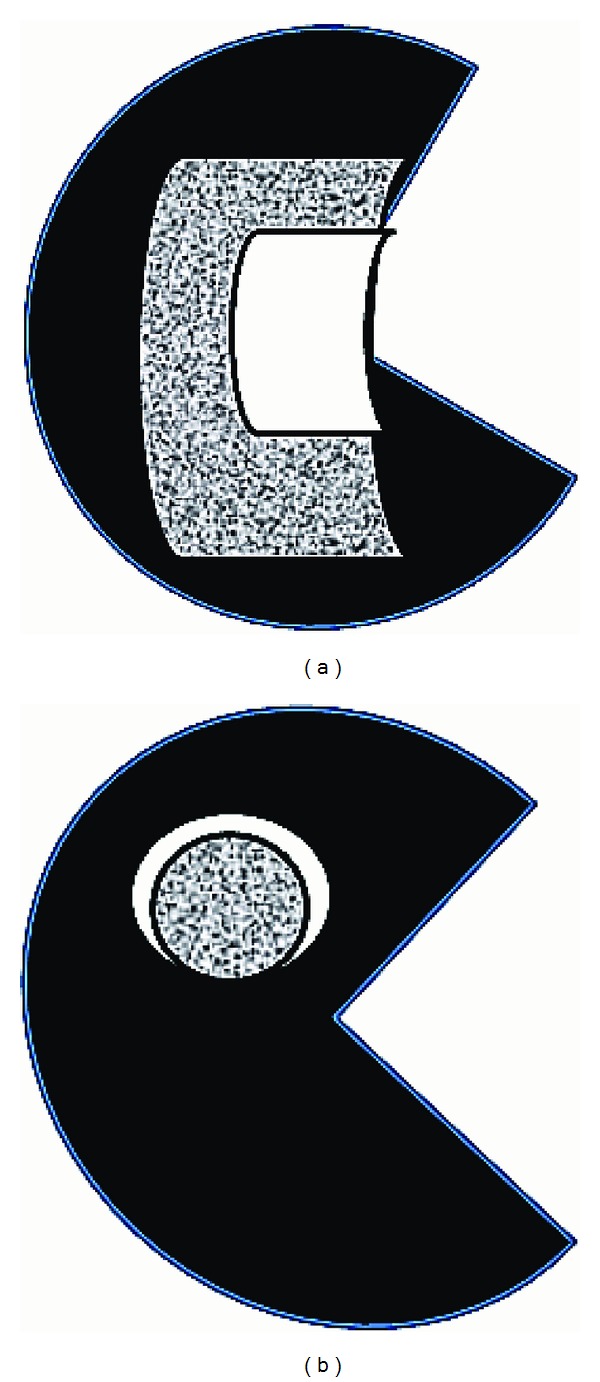
Comparison between myocardial edema (white) and scarring (dotted) in the myocardium. (a) At the acute stage of ischemic or inflammatory cardiomyopathy, myocardial edema is associated with smaller myocardial scarring or its absence. (b) In some types of nonischemic cardiomyopathy, myocardial edema is smaller than the scarring, indicating relapsed ischemia.

At the acute stage of ischemic or inflammatory cardiomyopathy, myocardial edema is larger than myocardial scarring seen in delayed-enhancement MRI, because the edema may surround inflammatory or dying tissues [10, 11]. When the myocardial edema is smaller than the scarring, the edema may reflect relapsed ischemia (Figures 5, 7, and 8). In some cardiomyopathy, including takotsubo cardiomyopathy, myocardial edema without scarring may give a clue of the diagnosis and suggest the good prognosis (Figure 8).

### 3.3. Artifacts

**Figure 9:**
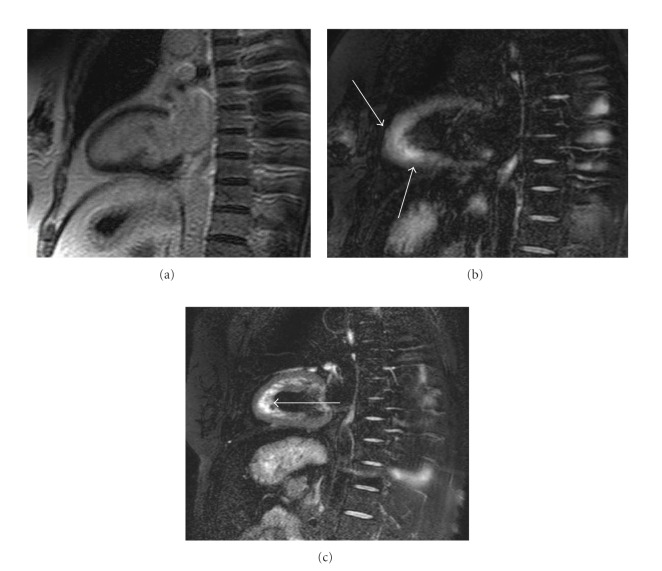
55-year-old female with takotsubo cardiomyopathy. Long-axis delayed enhancement (a) shows no myocardial scarring of the apical myocardium, whereas the T2-weighted MRI (b) shows circumferential myocardial edema at the apical and midventricular regions (arrows). The T2-weighted MRI at its remission (c) shows no myocardial edema. The flow artifact is seen in the apical cavity (arrow), due to the insufficient blood signal suppression by black-blood prepulse.

The intraventricular flow close to the hypokinetic myocardium is not nullified sufficiently (Figure 9(a)). Arrhythmia often impairs the image quality of cardiac MRI. Motion artifacts are another concern in patients with deteriorated conditions [13]. Incomplete shimming and magnetic inhomogeneity may lead to incomplete fat suppression or unwanted water suppression in the fat-suppressed T2-weighted MRI.

## 4. T2-Wieghted Cardiac MRI of Edema in Myocardial Diseases

### 4.1. Myocardial Infarction

Myocardial edema distributes to the coronary artery supply in acute myocardial infarction [10, 11]. T2-weighted MRI is useful for differentiating between acute and chronic myocardial infarction (Figure 2) [10, 11, 20]. The T2-weighted imaging is also valuable for the visualization of the area at risk that can be salvaged by appropriate intervention. However, Abdel-Aty et al. [21] have reported that myocardial edema in acute myocardial infarction may parallel systolic dysfunction and worsen the prognosis of the patients even without myocardial scarring. 

### 4.2. Acute Myocarditis

In acute myocarditis, myocardial edema is usually observed in the lateral wall (Figure 3) [11, 19]. The myocardial edema localizes in the subepicardial region dominantly and shows noncoronary distribution. The myocardial edema may be more extensive than myocardial hyperenhancement at the acute phase of this disease [12, 19]. T2-value calculation or mapping may be useful for the detection of diffuse myocardial edema associated with acute myocarditis. 

### 4.3. Eosinophilic Myocarditis

 In eosinophilic myocarditis, myocardial edema appears patchy or diffuse [22]. Churg-Strauss disease is a relapsing allergic disease, and the myocardial edema is patchy (Figure 4) and may be smaller than the myocardial scarring. Vasculitis, infiltration of the myocardium by eosinophils, and extensive edema characterize eosinophilic myocarditis induced by other etiologies. 

### 4.4. Sarcoidosis

Patchy myocardial edema is occasionally observed in cardiac sarcoidosis [12]. The myocardial edema localizes dominantly in the subepicardial region, and in the subendocardial or mesocardial myocardium (Figures 5 and 6(a)). The myocardial edema can induce ventricular arrhythmia or conduction disturbance, but responds to steroid therapy. The myocardial edema may be consistent with the myocardial inflammation and abnormal metabolism shown by ^18^FDG-PET (Figure 6(b)). Scarred myocardium does not respond to steroid therapy (Figure 5(a)). 

### 4.5. Hypertrophic Cardiomyopathy

Patchy mesocardial edema is often observed in hypertrophic cardiomyopathy (Figure 7(b)). The myocardial edema may reflect the myocardial ischemia and is related to chest pain or ischemic pattern on ECG in hypertrophic cardiomyopathy [23]. The myocardial edema can be smaller than or equal to the myocardial scarring in hypertrophic cardiomyopathy (Figure 7).

### 4.6. Takotsubo Cardiomyopathy

 Takotsubo cardiomyopathy is a reversible cardiomyopathy that occurs following a stressful event. This disease affects postmenopausal women, and the clinical and ECG findings are similar to those of myocardial infarction. However, takotsubo cardiomyopathy does not show myocardial scarring on delayed-enhancement MRI (Figure 9(a)). T2-weighted cardiac MRI shows the circumferential edema of the apical to midventricular myocardium, which matches regional dysfunction (Figure 9(b)) [24]. This distribution of myocardial edema with no or subtle delayed enhancement offers a clue for distinguishing takotsubo cardiomyopathy from myocardial infarction and myocarditis (Figures 2 and 9). The myocardial edema diminishes without any myocardial scarring and dysfunction at the remission (Figure 9(c)).

### 4.7. Following Interventional Procedures

**Figure 10:**
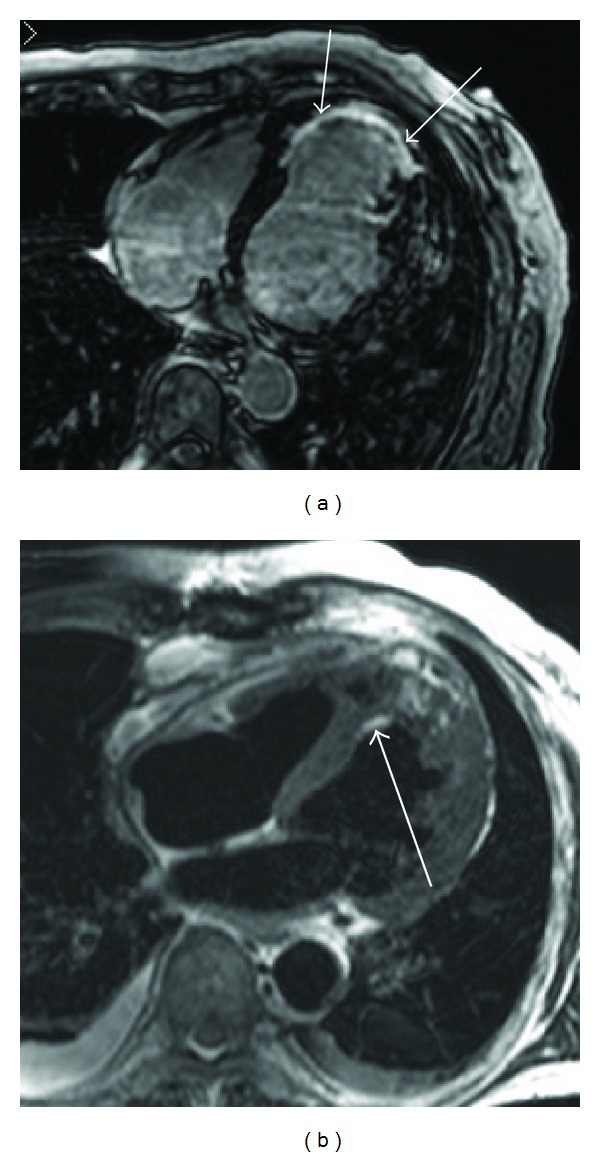
66-year-old male who has undergone cryoablation for ventricular tachycardia associated with chronic myocardial infarction. Delayed-enhancement MRI (a) visualizes apical myocardial scarring with various transmurality (arrows). T2-weighted MRI (b) shows myocardial edema after ablation (arrow), where nontransmural scarring has been seen in the delayed-enhancement MRI.

T2-weighted MRI is useful for the detection of myocardial edema after ablation therapy for ventricular arrhythmia associated with some cardiomyopathies. These patients tend to receive repeated ablations for arrhythmogenic myocardial scarring. T2-weighted MRI distinguishes the recently ablated region with edema from the myocardial scarring or previously ablated region (Figure 10).

## 5. Summary

T2-weighted cardiac MRI of edema is acquired in combination with acceleration, motion suppression, and other techniques. T2-weighted cardiac MRI visualizes myocardial edema that corresponds to ischemia, active inflammation, vasculitis, or recently performed intervention in the myocardium and provides information complementary to delayed-enhancement MRI.
